# Ferroptosis in lung cancer: a novel pathway regulating cell death and a promising target for drug therapy

**DOI:** 10.1038/s41420-023-01407-z

**Published:** 2023-04-01

**Authors:** Nan Xing, Qinyun Du, Sa Guo, Gelin Xiang, Yi Zhang, Xianli Meng, Li Xiang, Shaohui Wang

**Affiliations:** 1 State Key Laboratory of Southwestern Chinese Medicine Resources, School of Pharmacy, Chengdu University of Traditional Chinese Medicine, Chengdu, 611137 China; 2 State Key Laboratory of Southwestern Chinese Medicine Resources, School of Ethnic Medicine, Chengdu University of Traditional Chinese Medicine, Chengdu, 611137 China; 3 State Key Laboratory of Southwestern Chinese Medicine Resources, Meishan Hospital of Chengdu University of Traditional Chinese Medicine, Meishan, 620010 China; 4 State Key Laboratory of Southwestern Chinese Medicine Resources, Innovative Institute of Chinese Medicine and Pharmacy, Chengdu University of Traditional Chinese Medicine, Chengdu, 611137 China

**Keywords:** Lung cancer, Cell death

## Abstract

Lung cancer is a common malignant tumor that occurs in the human body and poses a serious threat to human health and quality of life. The existing treatment methods mainly include surgical treatment, chemotherapy, and radiotherapy. However, due to the strong metastatic characteristics of lung cancer and the emergence of related drug resistance and radiation resistance, the overall survival rate of lung cancer patients is not ideal. There is an urgent need to develop new treatment strategies or new effective drugs to treat lung cancer. Ferroptosis, a novel type of programmed cell death, is different from the traditional cell death pathways such as apoptosis, necrosis, pyroptosis and so on. It is caused by the increase of iron-dependent reactive oxygen species due to intracellular iron overload, which leads to the accumulation of lipid peroxides, thus inducing cell membrane oxidative damage, affecting the normal life process of cells, and finally promoting the process of ferroptosis. The regulation of ferroptosis is closely related to the normal physiological process of cells, and it involves iron metabolism, lipid metabolism, and the balance between oxygen-free radical reaction and lipid peroxidation. A large number of studies have confirmed that ferroptosis is a result of the combined action of the cellular oxidation/antioxidant system and cell membrane damage/repair, which has great potential application in tumor therapy. Therefore, this review aims to explore potential therapeutic targets for ferroptosis in lung cancer by clarifying the regulatory pathway of ferroptosis. Based on the study of ferroptosis, the regulation mechanism of ferroptosis in lung cancer was understood and the existing chemical drugs and natural compounds targeting ferroptosis in lung cancer were summarized, with the aim of providing new ideas for the treatment of lung cancer. In addition, it also provides the basis for the discovery and clinical application of chemical drugs and natural compounds targeting ferroptosis to effectively treat lung cancer.

## Facts

- Ferroptosis is a type of programmed cell death that differs from the traditional cell death pathways such as apoptosis, necrosis, pyroptosis etc.
- The regulatory mechanism of ferroptosis is mainly related to iron metabolism, lipid metabolism and redox dynamic equilibrium.
- Ferroptosis has considerable research value and application prospect for the treatment of lung cancer.
- It is a promising direction for the future treatment of lung cancer to find chemical drugs and natural compounds that target ferroptosis.

## Open questions

- What are the characteristics of ferroptosis?
- What is the link between ferroptosis and lung cancer?
- What are the chemical and natural drugs that target ferroptosis? How do they modulate ferroptosis and thus exert their anti-lung cancer effects?

## Introduction

Lung cancer is a common malignant tumor occurring in the human body, mainly originating in the trachea, bronchus, and lung. It is mainly divided into squamous cell carcinoma, adenocarcinoma, small cell carcinoma, and large cell carcinoma. According to the Global Cancer Statistics 2020, lung cancer is not only the most commonly diagnosed cancer after breast cancer, but also the leading cause of cancer death [1], In China, the death rate of lung cancer has remained the first from 2015 to 2020 [2, 3]. However, the cause of lung cancer is not completely clear up to now, it is mostly related to living habits, environment, heredity, and many other factors [4]. As there are no obvious pathological symptoms in the early stage of lung cancer, most of them are respiratory symptoms such as cough, dyspnea, chest pain, etc. [5], which leads to lung cancer patients easy to miss the optimal treatment time. At present, common treatments for lung cancer include surgery, radiotherapy, chemotherapy, and immunotherapy [6]. However, due to the special invasiveness of lung cancer [7, 8], as well as the dependence of appellate therapy on patients’ physical conditions and family conditions, the prognosis of lung cancer is poorly treated. At the same time, with the emergence of resistance of lung cancer cells to some therapeutic means and the emergence of different degrees of resistance to a variety of therapeutic drugs, the therapeutic effect of lung cancer is often unsatisfactory. Therefore, it is urgent to find and explore new therapeutic drugs and targets for lung cancer [9, 10].

Programmed cell death (PCD) is a cell death process mediated by molecular programs regulated by specific genes in cells, and plays an important role in the normal development process and homeostasis maintenance of multicellular organisms. To date, the most well-studied PCD includes apoptosis, necroptosis, pyroptosis, ferroptosis, and autophagy, among others [11]. Ferroptosis is a novel form of PCD first proposed in 2012, which is regulated by genes in the same way as many PCD modalities and results from the interaction of multiple cellular signaling pathways [12]. Its characteristics mainly include smaller mitochondria, increased membrane density, reduced cristae, no obvious morphological changes in the nucleus, increased lipid peroxidation, increased ROS, and changes in some characteristic genes (such as GPX4, TFR1, etc.) [13]. However, it differs from other forms of PCD in that ferroptosis is based on divalent iron and leads to cell death by promoting lethal accumulation of intracellular lipid peroxides [12]. Interestingly, cancer cells are more iron-addicted than normal cells, because iron is one of the essential elements of the body. These cancer cells need more iron during the production process, and the imbalance of iron metabolism can increase the risk of cancer and promote tumor growth [14]. As a consequence, by promoting ferroptosis in lung tumors, the development of lung cancer can be inhibited, and at the same time, the drug resistance and radiation resistance of lung cancer can be resisted to a certain extent [15]. And this may be just right to become a breakthrough in the current problem of lung cancer treatment facing. Therefore, this review will further explore the potential therapeutic targets of ferroptosis in lung cancer and summarize the existing drugs for the treatment of ferroptosis in lung cancer, in order to provide a theoretical basis for further research of ferroptosis in lung cancer.

## Ferroptosis

**Fig. 1:**
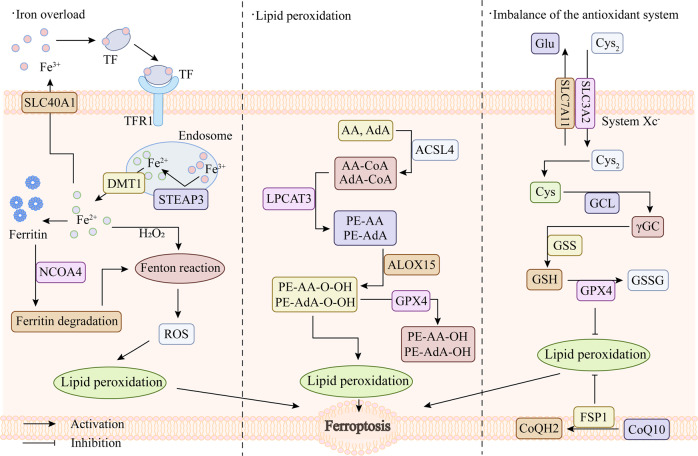
Core regulatory mechanisms of ferroptosis. TF transferrin, TFR1 transferrin receptor, SLC40A1 solute carrier family 40 member 1, DMT1 solute carrier family 11 member 2, STEAP3 STEAP3 metalloreductase, NCOA4 nuclear receptor coactivator 4, ROS reactive oxygen species, AA arachidonic acid, Ada adrenic acid, CoA coenzyme A, PE phosphoethanolamine, PE-OOH phosphatidylethanolamine hydroperoxide, PE-OH alcohol phospholipid hydroxide, ACLS4 acyl-CoA synthetase long chain family member 4, LPCAT3 lysophosphatidylcholine acyltransferase 3, ALOX15 arachidonate 15-lipoxygenase, GPX4 glutathione peroxidase 4, System Xc^−^ cystine/glutamate antiporter system, Cys_2_ cystine, Glu glutamate, Cys cysteine, Gly glycine, SLC3A2 solute carrier family 3, member 2, SLC7A11 solute carrier family 7 (L-type amino acid transporter), member 11, GCL glutamate-cysteine ligase, γGC gamma-glutamylcysteine, GSS glutathione synthetase, GSH glutathione, GSSG oxidized glutathione, CoQ10 coenzyme Q10, FSP1 ferroptosis inhibitor protein 1, CoQH2 ubiquinol.

Cell death is an inevitable and irreversible process of life in the body [16, 17]. The existing cell death pathways include apoptosis, necroptosis, autophagy, ferroptosis, pyroptosis, and necrosis [18]. The concept of ferroptosis was first proposed in 2012 by Scott J Dixon et al., and it is iron-dependent and distinct from other forms of cell death [12]. When ferroptosis occurs, there is no cell shrinkage, chromatin condensation, formation of apoptotic bodies and disintegration of the cytoskeleton, which are the traditional phenomena of apoptosis [19, 20]. It is mainly manifested as mitochondrial morphological changes under the electron microscope, such as mitochondrial shrinkage, cristal reduction, membrane density increase, outer membrane rupture, etc. [12, 13]. The biochemical characteristics of ferroptosis are mainly manifested as the accumulation of intracellular iron, the production of reactive oxygen species (ROS), the increase of lipid peroxides, and the decreased expression of glutathione peroxidase 4 in antioxidant system (glutathione system) [21]. The regulatory mechanism of ferroptosis is mainly related to iron metabolism, lipid metabolism and redox dynamic equilibrium (Fig. 1).

### Iron overload

As a common trace element, iron is distributed in the liver, spleen and bone marrow of the human body, which is closely related to the hematopoietic function of body and is necessary to maintain the normal life activities of human [22, 23]. Most iron in the body is obtained from food and then absorbed by active transport in the duodenum and upper quarter of the small intestine. The absorbed Fe^2+^ is oxidized to Fe^3+^ in epithelial cells of small intestine, which is then bound to transferrin (TF) in plasma and transported to various sites of utilization or storage [24–26]. Iron is involved in oxygen transport, electron transfer reaction in mitochondria, DNA synthesis and other processes [27]. And all of these processes are based on iron homeostasis [28]. Conversely, when one of the components of iron uptake, storage, utilization and efflux is interfered [29], such as increased degradation of ferritin induced by nuclear receptor coactivator 4 (NCOA4) or inhibition of the iron export protein SLC40A1 [30, 31], intracellular iron homeostasis will be affected. Subsequently, with the increase of Fe^2+^, it will react with hydrogen peroxide (H_2_O_2_) in a Fenton reaction and produce large amounts of ROS, which is harmful to cells [32, 33], thereby breaking the redox balance in cells and causing oxidative stress [34]. Under normal circumstances, oxygen-free radical reaction and lipid peroxidation reaction are in a state of coordination and dynamic balance. When excessive ROS occurs, the dynamic balance of the two will be disordered and dysregulated, which will cause metabolic disorders and decreased immune function, form a chain reaction of oxygen free radicals, start lipid peroxidation reaction, and thus cause ferroptosis of cells [35, 36].

### Lipid peroxidation

The cell membrane is mainly composed of lipids, proteins, and sugars, which are essential for maintaining intracellular and extracellular stability, regulating the movement of substances into and out of cells, and conducting intercellular signal transduction [37]. Lipid peroxide (LPO) is a product of the reaction of oxygen free radicals with polyunsaturated fatty acids (PUFA), and it is present in extremely low amounts under normal conditions [38]. However, when intracellular ROS increases, it will combine with the polyunsaturated fatty acid side chains of phospholipids, enzymes or membrane receptors, and lipid peroxidation will occur to generate large amounts of LPO, which will destroy the normal fluidity and permeability of cell membrane, leading to changes in cell structure and function, and then induce cell death [39, 40]. Arachidonic acid lipoxygenase 15 (ALOX15), acyl-coa synthetase long-chain family member 4 (ACSL4) and lysophosphatidylcholine acyltransferase 3 (LPCAT3) are key enzymes in lipid metabolism and are required for ferroptosis. Acyls-arachidonoyl (AA) and adrenoyl (AdA) were firstly catalyzed by ACSL4 to generate acyl Co-A derivatives [41, 42]. LPCAT3 then esterified these derivatives to phosphatidylethanolamines (AA-PE and AdA-PE) [43]. Lipoxygenases (LOX) are a family of iron-containing enzymes [44], which can oxidize PUFA by phosphorylating enzyme kinase G2 (PHKG2) -dependent iron pools through the diallyl site [45]. Afterwards, AA-PE and AdA-PE catalyzed by LPCAT3 are oxidized by ALOX15 to generate LPO, leading to ferroptosis [46]. In addition, some studies have shown that cytochrome P450 reductase (POR) and cytochrome B5 reductase (CYB5R1) can also promote ferroptosis by catalyzing phospholipid peroxidation. POR may promote lipid peroxidation by accelerating the cycling between Fe^2+^ and Fe^3+^ in heme [47]. Meanwhile, POR and CYB5R1 can also transfer electrons from NAD(P)H to oxygen to generate hydrogen peroxide, which subsequently generates reactive hydroxyl radicals through the Fenton reaction and further promotes the production of LPO, destroying the integrity of the cell membrane [48]. However, the specific mechanism of action is still unclear and needs to be further explored.

### Imbalance of the antioxidant system

Glutathione (GSH), a tripeptide containing sulfhydryl group consisting of glutamate (Glu), cysteine (Cys) and glycine (Gly), has antioxidant and free radical scavenging ability [49]. The cystine/glutamate antiporter system (system x_c_^−^) is an amino acid transporter expressed at the plasma membrane and consists of a light chain, xCT, and a heavy chain, 4F2 [50]. It can export Glu and import cystine (Cys_2_) in a 1:1 ratio, then reduce intracellular Cys_2_ to Cys and further synthesize GSH [51]. Glutathione peroxidase 4 (GPX4) is a key enzyme in the glutathione antioxidant system. It can use GSH as a substrate to convert LPO into lipid alcohols that are harmless to the cell membrane, thereby inhibiting the lipid peroxidation caused by the increase of ROS [52, 53]. However, the generation of substrate GSH is tightly regulated by system x_c_^−^. When Cys_2_ is deprived and Cys uptake is inhibited, GSH conversion is insufficient, which allows lipid peroxidation products to accumulate and lead to ferroptosis [54, 55]. However, inhibiting GPX4, inhibiting system x_c_^−^, or blocking GPX4 synthesis can all affect the intracellular redox balance, reduce the antioxidant capacity of cells, and reduce the elimination of lipid peroxides [56, 57]. At the same time, non-canonical GPX4 regulatory pathways can also regulate the occurrence of ferroptosis. Studies have shown that ferroptosis inhibitor protein 1 (FSP1) has a parallel effect with GPX4, which can reduce coenzyme Q10 (CoQ10) to ubiquinol (CoQH2) on the cell membrane. Then CoQH2 inhibits ferroptosis by trapping free radicals and acting as an antioxidant [58, 59]. Moreover, FSP1 can also reduce vitamin K to hydroquinone to play a role in trapping free radicals, thereby protecting cells from ferroptosis [60]. Further studies showed that mitochondrial dihydroorotate dehydrogenase (DHODH) also runs in parallel with mitochondrial GPX4 to oxidify dihydroorotate to orotate and reduce panquinol to pantanol, thus acting as an anti-ferroptosis protein in the inner mitochondrial membrane [61]. In addition, tetrahydrobiopterin (BH4), which is produced by guanosine triphosphate (GTP) conversion and synthesized using GTP cyclohydrolase 1 (GCH1) as the rate-limiting enzyme, has also been shown to possess endogenous antioxidant effects [62]. BH4 can act against ferroptosis by selectively preventing the depletion of phospholipids with two polyunsaturated fatty acyl groups [63, 64]. Therefore, with the deepening of research, the regulatory pathways of ferroptosis in the antioxidant system are not limited to GSH-dependent antioxidant pathways, but also can play a role in regulating ferroptosis through GSH-independent antioxidant pathways, which means that there may be more regulatory mechanisms related to ferroptosis waiting for us to further explore.

## Targets of ferroptosis in lung cancer

**Fig. 2:**
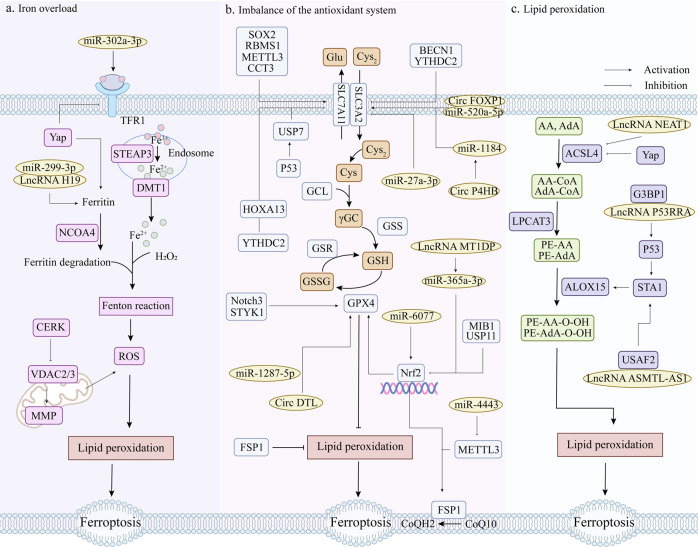
Relevant targets and their interactions for targeting ferroptosis for lung cancer therapy. GPX4 glutathione peroxidase 4, SLC3A2 solute carrier family 3, member 2, SLC7A11 solute carrier family 7 (L-type amino acid transporter), member 11, RBMS1 RNA-binding protein, SOX2 stem cell transcriptional factor, AKR1B1 aldo-keto reductase family 1 member B1, STAT3 activator of transcription 3, METTL3 methyltransferase-like 3, CCT3 chaperonin containing TCP1 subunit 3, Nrf2 nuclear factor erythroid 2-related factor 2, GCL glutamate-cysteine ligase, γGC gamma-glutamylcysteine, MIB1 E3 ubiquitin ligase, p53 tumor suppressor protein, SAT1 spermidine/spermine N1-acetyltransferase 1, GLS2 glutaminase 2, FSP1 ferroptosis inhibitor protein 1, NFS1 NFS1 cysteine desulfurase, STYK1 Serine-threonine tyrosine kinase 1, LSH Lymph-specific helicase, GINS4 a member of GINS family proteins, Yap Yes associated transcriptional regulator, TFR1 transferrin receptor, FTH ferritin heavy chain, FTL ferritin light chain, BECN1 ecombinant human autophagy effector protein, USP7 ubiquitin-specific processing protease 7, USP11 ubiquitin-specific processing protease 11, CRL4^DCAF8^ cullin-RING E3 ligase complex, YTHDC2 N6-methyladenosine (m6A) RNA binding protein, A20 ubiquitin-regulated enzyme, SENP1 Small ubiquitin-like modifier specific protease 1, Notch3 the third subtype of Notch family, PRDX6 peroxiredoxin 6, CERK ceramide kinaseCeramide kinase, VDAC voltage-dependent anion-selective channel, MMP mitochondrial membrane potential, miRNA MicroRNA, circRNA Circular RNA, lncRNA Long non-coding RNA, METTL3 methyltransferase-like 3, FABP3 fatty acid binding protein 3, ELAVL1 RNA-binding protein, CBS cystathionine β-synthase, G3BP1 Ras GTPase activating protein binding protein 1.

Targeting ferroptosis in lung cancer can inhibit the development process and metastasis of lung cancer, and also overcome the drug resistance and radiation resistance of lung cancer cells to a certain extent. According to the current research content, the targets of ferroptosis in lung cancer mainly focus on the regulation of antioxidant pathways, among which GPX4 and SLC7A11 are the core targets of the GSH-dependent antioxidant pathway. In addition, many studies have suggested that many non-coding RNAs play an important role in the regulation of ferroptosis in lung cancer. Furthermore, ferroptosis in lung cancer is also closely related to autophagy and ubiquitin-proteasome pathways. Therefore, we sorted out the relevant targets of ferroptosis in lung cancer therapy and their interactions, as shown in Fig. 2.

### GPX4

GPX4, also known as phospholipid hydroperoxide glutathione peroxidase (PHGPx), is the fourth member of the selenium-containing GPX family and is capable of removing lipid peroxides that accumulate in cells [65]. Due to its potent antioxidant activity, GPX4 exerts a negative effect on ferroptosis in a variety of cancer cells. For example, when GPX4 is overexpressed, HT-1080 cells are resistant to RSL3, whereas the knockdown of GPX4 makes HT-1080 cells sensitive to RSL3 [53]. In the study of lung cancer, the immunohistochemical staining results of clinical lung adenocarcinoma samples compared with normal alveolar epithelial cells and bronchial epithelial cells showed that GPX4 was strongly positive in lung adenocarcinoma tissues. And by targeting GPX4 can induce ferroptosis in lung adenocarcinoma cells and reverse their resistance to epidermal growth factor receptor-tyrosine kinase inhibitor (EGFR-TKI) [66]. Meanwhile, targeting GPX4 can also promote lapatinib (Lap) -induced ferroptosis in drug-resistant non-small cell lung cancer cells [67]. In addition to lapatinib, GPX4 was also positively correlated with chemoresistance to anticancer drugs L-685458, palbociclib, and topotecan [68, 69]. At the same time, inhibition of GPX4 expression can partially reduce the radiation resistance of lung cancer cells and promote the efficacy of radiotherapy [70]. In conclusion, GPX4 can affect the development of lung cancer by regulating ferroptosis. On the other hand, it is also related to the drug resistance and radiation resistance of lung cancer, and has great significance in the research of ferroptosis in lung cancer.

### SLC7A11

Cystine transporter solute carrier family 7 member 11 (SLC7A11) encodes the light chain subunit xCT of system x_c_^−^ and promotes GSH synthesis by mediating Cys uptake and Glu release [51, 71]. SLC7A11 is overexpressed in multiple human cancers and promotes tumor survival [72–74]. Study has shown that controlling H3K9me3 demethylation in the promoter region of SLC7A11 can increase SLC7A11 expression, inhibit ferroptosis, and promote osteosarcoma cell lung metastasis [75]. Through RNAseq and miRNAseq data analysis of lung adenocarcinoma (LUAD) in the Cancer Genome Atlas (TCGA) data, it was found that SLC7A11 is a potential biomarker for the prognosis of patients with LUAD [76]. Further studies showed that SLC7A11 regulated ferroptosis in lung cancer mainly by regulating metabolic demand [77]. An RNA-binding protein, RBMS1, interacts with eIF3d to bridge the 3’- and 5’-UTR of SLC7A11, facilitating its translation. When RBMS1 was ablated, SLC7A11 translation was inhibited and ferroptosis was induced [78]. Moreover, SLC7A11 expression is positively correlated with the stem cell transcription factor SOX2, which showed a high binding affinity for sequences with CTTTGTT located on the SLC7A11 promoter. When SOX2 is overexpressed, endogenous SLC7A11 expression is enhanced at both mRNA and protein levels. This will maintain a certain level of Cys in the cells to promote ferroptosis resistance of lung cancer cells [79]. It has also been shown that aldo-keto reductase family 1 member B1 (AKR1B1) interacts with signal transducer and activator of transcription 3 (STAT3) to up-regulate SLC7A11, which leads to increased Cys_2_ uptake in lung cancer cell lines and xenograft mouse models, and further promotes lung cancer cell resistance [80]. Methyltransferase-like 3 (METTL3) can directly target the m6 modification of SLC7A11, which enables m6A reading protein YTHDF1 to recognize the mRNA of SLC7A11, and then promote the expression of SLC7A11 by enhancing its stability and translation, thereby inhibiting ferroptosis [81]. Meanwhile, the chaperone protein containing TCP1 subunit 3 (CCT3) can also inhibit ferroptosis by promoting SLC7A11 expression in lung cancer cells [82]. In conclusion, the inhibition of ferroptosis caused by SLC7A11 up-regulation is very unfavorable for the treatment of lung cancer, but it also reflects its important regulatory role in the ferroptosis of lung cancer. Therefore, it is very beneficial to find more upstream targets of SLC7A11 regulation to promote ferroptosis of lung cancer.

### Nrf2

Nuclear factor erythroid 2-related factor 2 (Nfr2), a member of the Cap’n’Collar (CNC) subfamily of transcription factors, is a key factor in cellular oxidative stress [83]. Under normal conditions, Keel-like ECH-associated protein 1 (KEAP1) binds to Nrf2 and undergoes ubiquitin-mediated degradation to maintain the Nrf2 level at a low level [84]. However, when cells are exposed to oxidative stress, the nuclear accumulation and constitutive activation of Nrf2 will increase, which will promote cancer progression and is also an important cause of tumor drug resistance [85, 86]. Inhibition of Nrf2 expression or activity can increase the sensitivity of tumors to anticancer drugs [87]. It also has a strong correlation with ferroptosis in lung cancer. Study has shown that Nrf2 is highly expressed in cancer tissues of NSCLC patients and can regulate the sensitivity of NSCLC cells to ferroptosis induced by Cys_2_ deprivation [88]. Nrf2 promotes γ-glutamyl-peptide (γGC) synthesis and inhibits ferroptosis caused by Cys_2_ deficiency by regulating the catalytic subunit of glutamate-cysteine ligase (GCLC) [89]. It can also promote chemoresistance of non-small cell lung cancer brain metastases by targeting GPX4 and binding to the GPX4 promoter region [90]. When Nrf2 is degraded by E3 ubiquitin ligase (MIB1), the antioxidant capacity of lung cancer cells is weakened, and the sensitivity of lung cancer cells to ferroptosis inducers is enhanced [91]. Therefore, inhibiting Nrf2 or promoting its degradation may be beneficial for ferroptosis induction in lung cancer.

### ACSL4

Acyl-CoA synthetase long-chain family member 4 (ACSL4), a member of the long-chain acyl-CoA synthetase (ACS) family, is involved in the regulation of arachidonic acid (AA) and eicosapentaenoic acid [41]. ACSLA can specifically esterify arachidonic acid (AA) and epinephrine (AdA) to phosphatidyleolamine (PE) [92]. Based on lipidomics analysis, activated ACSL4 can catalyze the synthesis of lipid organisms containing polyunsaturated fatty acids (PUFA), leading to the accumulation of lipid peroxides, which in turn promotes ferroptosis [93]. When ACSL4 is highly expressed, it can improve the sensitivity of breast cancer to neoadjuvant chemotherapy [94]. Is it also a potential therapeutic target for ferroptosis in lung cancer? Through TCGA database analysis and immunohistochemical staining, ACSL4 is down-regulated in lung adenocarcinoma. When ACSL4 is knocked down in vitro, the survival rate and invasiveness of lung cancer cells are enhanced, on the contrary, overexpression would promote ferroptosis of lung cancer cells [95]. In addition, ACSL4 was positively correlated with erastin-induced ferroptosis in NSCLC cells [96]. When ACSL4 is upregulated, lipid peroxidation products and lethal reactive oxygen species accumulate, thus sensitiating lung cancer cells to ferroptosis [97, 98]. Therefore, targeting ACSL4 is an effective way to promote ferroptosis in lung cancer.

### P53

P53 is a tumor suppressor, and the cell signal transduction pathway mediated by p53 plays a crucial role in regulating the normal life activities of cells [99]. Activation of p53 could not directly induce ferroptosis, but it can inhibit the expression of SLC7A11 by promoting nuclear translocation of the deubiquitinase USP7 and then decreasing the occupancy of monoubiquitination of histone H2B on lysine 120 (H2Bub1) in the regulatory region of SLC7A11 gene [100]. Alternatively, P53 enhances the sensitivity of cells to ferroptosis by enhancing the expression of spermidine/spermine N1-acetyltransferase 1 (SAT1) and glutaminase 2 (GLS2) and inhibiting cystine uptake [101–103]. Levobupivacaine, a local anesthetic, induces ferroptosis in NSCLC cells by upregulating p53 [104]. Meanwhile, erastin also inhibited the corresponding target gene SLC7A11 by activating p53 to promote ROS accumulation [105]. Furthermore, some researchers have used nano-cascade engineering to construct metal-organic supermolecules with p53-activating peptides and CeO_2_ nanoparticles, which can activate the p53 signaling pathway and down-regulate the downstream gene GPX4 in vitro and in vivo, thereby inhibiting the development and progression of lung cancer [106]. Therefore, the ferroptosis-promoting effect of targeting p53 in lung cancer should be well utilized.

### FSP1

Inhibitor of ferroptosis 1 (FSP1), also known as flavoprotein apoptosis-inducing factor mitochondria-associated 2 (AIFM2), is a glutathione-independent inhibitor of ferroptosis [107]. FSP1 can use reduced coenzyme II (NAD(P)H) to catalyze the regeneration of ubiquinone (CoQ10) and inhibit ferroptosis by reducing ubiquinone to panthol and capturing free radicals that mediate lipid peroxidation [59]. There is a positive correlation between the expression of FSP1 and ferroptosis resistance of lung cancer cells and xenografts [58]. A recent study identified FSP1 as a transcriptional target of Nrf2. When the CoQ-FSP1 axis is inhibited, the sensitivity of KEAP1-deficient lung cancer cells and patient-derived xenograft tumors to radiation is enhanced [108]. And a plasma-activated medium (PAM) also promotes cell death by depleting FSP1 in human lung cancer cells and increasing intracellular lipid ROS [109]. Therefore, FSP1 has a good application prospect in the ferroptosis of lung cancer. Inhibition of FSP1 expression or depletion of FSP1 can promote ferroptosis of lung cancer.

### NFS1

Iron-sulfur cluster synthase (NFS1) is an essential enzyme in eukaryotes to obtain sulfur from Cys for the synthesis of iron-sulfur clusters (ISC) [110]. Study has shown that when ISC synthesis is reduced, iron load is increased, making cancer cells sensitive to ferroptosis [111]. Inhibition of NFS1, which is required for primary lung tumor growth, limits the availability of ISC and strongly activates the iron deficiency response [112]. More recently, it has been suggested that NFS1 inhibition may act synergistically with glutathione (GSH) synthesis inhibition to promote cell death through ferroptosis [113]. An anti-cancer drug, Eprenetapopt, inhibits ISC synthesis by inhibiting cysteine desulfurase activity, thereby inducing ferroptosis and limiting cell proliferation [114]. In conclusion, affecting the synthesis of ISC by inhibiting NFS1 and thus altering the sensitivity of cells to ferroptosis is a potential therapeutic tool to promote ferroptosis in lung cancer, but the precise mechanism remains to be further investigated.

### STYK1/NOK

Serine-threonine tyrosine kinase 1 (STYK1), also known as a novel oncogene with kinase domain (NOK), belongs to the receptor protein tyrosine kinases (RPTKs) subfamily [115], and has a strong ability to promote tumor formation and metastatic capacity [116]. Studies have found that NOK is positively expressed in tumor cells of NSCLC patients, and it is significantly correlated with the degree of lung tumor differentiation and lymphatic metastasis [117]. When STYK1 is overexpressed, it promotes proliferation, migration, invasion, and epithelial-mesenchymal transition (EMT) of NSCLC cells [118]. Excitingly, the overexpression of STYK1 in lung cancer cell line SW900 upregulates GPX4 expression, promotes cell proliferation and attenuates ferroptosis-specific mitochondrial abnormalities, while downregulation of GPX4 exacerbates this attenuation without affecting cell proliferation [119]. In summary, the current study highlights that STYK1 expression is upregulated in NSCLC, that overexpression of STYK1 is associated with worse prognosis, that STYK1 is positively correlated with GPX4, and that overexpression of STYK1 inhibits ferroptosis in NSCLC cells, which provides a new opportunity to study the oncogenic mechanisms of STYK1.

### LSH

Lymphoid-specific helicase (LSH) is a member of the ATP-dependent chromatin remodeling protein SNF2 helicase family [120], which is involved in a variety of epigenetic regulation including nucleosome remodeling, DNA methylation, and histone modification [121]. LSH, as an oncogene, has been found to be highly expressed in lung cancer tissues, and is positively correlated with the expression of GINS4. Further studies have found that LSH can increase the expression of GINS4 by stabilizing its post-transcriptional mRNA level, thus promoting the progression of lung cancer [122]. In addition, LSH can activate genes related to glucose transporter (GLUTs) and fatty acid dessaturase (FADSs), and affect the metabolism of lung cancer cells. It has been demonstrated that LSH can interact with WDR76 to restrain ferroptosis through activating lipid metabolism-related genes (including GLUT1) and ferroptosis-related genes FADS2 and sterol-coenzyme A desaturase 1 (SCD1), and thus participate in the Warburg effect [123]. In short, the above studies provide ample evidence that LSH, as an oncogene, is participated in ferroptosis and is a possible therapeutic target for lung cancer due to its key effect in ferroptosis. However, current studies are still limited and more experiments are needed in the future to further demonstrate whether LSH has a regulatory effect on ferroptosis in lung cancer.

### Yap

Yes-associated protein (Yap) is the key transcription factor of the Hippo tumor suppressor pathway, which is closely related to cell growth, tissue homeostasis and organ size [124, 125]. Yap has a significant promoting effect on cancer progression, drug resistance and metastasis of NSCLC [126, 127]. On the contrary, reducing the expression or inhibiting the activity of Yap can inhibit the growth and metastasis of NSCLC tumors [128]. A recent study has shown that Yap is associated with the activation of ferroptosis, Yap can promote an iron environment in cancer cells through stimulating transcription of TEAF-based members of the Transferrin receptor (TFRC) and Acyl-CoA synthetase long chain family 4 (ACSL4) [129, 130]. Activation of the Hippo pathway can damage an iron environment. Surprisingly, the accumulate of endogenous glutamate after system x_c_^−^ inhibition can determine the sensitivity of ferroptosis by inhibiting Yap in LUAD cells. When GFPT1 is impaired, Yap O-GlcNAcylation and expression are not sustained in LUAD cells. As a result, Hippo pathway like ubiquitination and phosphorylation of YAP are strengthened [130]. Furthermore, Yap can also control intracellular iron levels by affecting the transcription of ferritin heavy chain (FTH) and ferritin light chain (FTL), thereby regulating cell ferroptosis sensitivity [131]. In a word, Yap can not only regulate tumor metastasis and progression on the one hand, but also affect ferroptosis on the other hand. The above studies demonstrate that YAP is crucial in regulating both the initiation and development of ferroptosis, and Yap can be a good candidate target for lung cancer therapy.

### The relationship with autophagy

Autophagy is a process that engulfs its own cytoplasmic proteins or organelles and encapsulates them into vesicles, then unites with lysosomes to remove the encapsulated contents [132]. Ferritin is very important for maintaining iron homeostasis in cells. Recent studies have shown that the occurrence of iron death is dependent on autophagy and that many iron death regulators are considered to be potential regulators of autophagy [133]. It has been reported that ROS generated by ferroptosis inducer erastin can induce autophagy, promote ferritin degradation, and induce TFR1 expression, thereby leading to ferroptosis [134]. Moreover, there is increasing evidence that ferroptosis is an autophagy-dependent form of cell death and that either intracellular transition activation or abnormally elevated lysosomal activity can cause the accumulation of intracellular iron ions and lipid peroxides, which in turn promotes ferroptosis. Particularly, ferritin phagocytosis promoted by NCOA4, BECN1-mediated system xc^−^ inhibition, lysosomal membrane penetration induced by STAT3, and chaperon-mediated autophagy of HSP90 related molecules can all play a role in promoting ferroptosis [135]. NCOA4 was identified as a cargo receptor that mediates ferritin phagocytosis and maintains intracellular iron homeostasis [136]. By directly binding to SLC7A11, recombinant human autophagy effector protein (BECN1) inhibits the activity of system x_c_^−^, promotes GSH consumption and lipid peroxidation, and induces ferroptosis in cancer cells [137]. In short, the above studies fully demonstrate that there is a connection between the regulation of ferroptosis and autophagy, and the multiple fine regulatory mechanisms between them will be gradually revealed in the future.

### The relationship with the ubiquitin-proteasome system

The ubiquitin-proteasome system (UPS) is an important regulatory system for protein degradation in cells. It can affect or regulate a variety of life activities in cells through polyubiquitination of substrate proteins and degradation by the proteasome [138]. There is growing evidence that that the dysregulation of UPS is related to the development and treatment of cancer, and these enzymes can interact with ferroptosis related proteins, thereby mediating the sensitivity of cancer cells to ferroptosis [139, 140]. Pyridinolone palladium complex (PdPT), a broad-spectrum deubiquitinase inhibitor, can promote the death of non-small cell lung cancer cells by inducing the degradation of GPX4 protein [141]. Ubiquitin specific protease 35 (USP35) can directly interact with ferroportin to maintain the stability of ferroportin and prevent iron overload. Knockdown of USP35 can inhibit the progression of lung cancer, promote ferroptosis, and increase the sensitivity of lung cancer cells to cisplatin and paclitaxel chemotherapy [142]. Ubiquitin-specific processing protease 11 (USP11) can interact with Nrf2 to deubiquitinate Nrf2. Conversely, depletion of USP11 inhibited H1299 cell proliferation and induced ferroptosis [143]. What’s more, cullin-RING E3 ligase complex CRL4^DCAF8^ regulates ferroptosis by controlling the stability of LSH [144]. In conclusion, targeting USP can indirectly regulate ferroptosis by controlling the stability of ferroptosis related proteins, which brings new opportunities and challenges for the treatment of lung cancer. However, due to the complexity of USP regulation, the precise mechanism of its interaction with ferroptosis is still unknown, which requires more future studies to confirm.

### Non-coding RNA

Non-coding RNAs refer to the RNAs transcribed from the genome, which can exercise their own biological functions without coding proteins. More and more studies have shown that non-coding RNAs play an important regulatory role in tumor development [145, 146]. In recent years, as researchers have deepened their research on ferroptosis, more and more non-coding RNAs have been proven to regulate ferroptosis in tumor cells [96, 147, 148].

MicroRNAs (miRNAs) are a group of endogenous non-coding RNAs that regulate gene expression, which can regulate the cell cycle, metastasis, angiogenesis, metabolism and apoptosis, and are considered as promising biomarkers in tumor diagnosis and disease prognosis [149, 150]. Many studies have shown that miRNAs are closely related to lung cancer drug resistance. Exosomal miR-4443 can reduce the expression level of methyltransferase-like 3 (METTL3) by targeting METTL3, and increase the level of FSP1 by N6-methyladenosine (m6A) methylation to inhibit ferroptosis and make NSCLC cells resistant to CDDP [151]. At the same time, miR-6077 could protect LUAD cells from cell death induced by CDDP combined with pemetrexed (PEM) by regulating the KEAP1-Nrf2-SLC7A11/NQO1 axis to mediate ferroptosis [147]. When miR-27a-3p is overexpressed, it directly binds to the 3’-UTR of SLC7A11, leading to its inhibition and subsequently reducing the ferroptosis induced by erastin. On the contrary, when miR-27a-3p is inhibited, NSCLC cells become more sensitive to erastin [152]. Moreover, by targeting ferroportin, miR-302a-3p can induce lipid peroxidation and iron overload, inhibit the growth and colony formation of lung cancer cells, and increase their sensitivity to CDDP and paclitaxel chemotherapy [153]. In addition, bioinformatics analysis has been used by some scholars to find that miR-17-5p may lead to ferroptosis-related brain metastasis in lung adenocarcinoma patients by down-regulating HOXA7 [154], but further experiments are lacking to verify this. Taken together, these results suggest that microRNAs may be involved in lung cancer drug resistance by regulating ferroptosis.

Circular RNAs (circRNAs) are a class of single-stranded non-coding RNAs that form circular conformations through non-canonical splicing or back-splicing events, and play a key role in the occurrence and development of a variety of tumors [155, 156]. Many circRNAs can act as microRNA sponges to relieve the inhibitory effect of micrornas on their target genes and increase the expression level of corresponding genes [157, 158]. Some studies have found that circ FOXP1 was significantly overexpressed in the serum of NSCLC patients [159], and the down-regulation of circ FOXP1 can inhibit the proliferation of lung cancer cells [160, 161]. Further studies showed that circ FOXP1 can increase the expression of SLC7A11 by directly sponging miR-520a-5p in lung cancer cells to promote lung cancer tumor growth [148]. Circ DTL is also up-regulated in NSCLC cells, which can play a carcinogenic role by targeting the miR-1287-5p/GPX4 axis. On the contrary, silencing circ DTL can promote the sensitivity of lung cancer cells to chemotherapy drugs and inhibit tumor growth in vivo [162]. And circ P4HB could regulate SLC7A11 by regulating miR-1184 to trigger GSH synthesis, which can protect lung cancer cells from erastin-induced ferroptosis, and promote tumor growth in vivo [163]. In addition, exosomal circRNA 101093 regulates overall arachidonic acid (AA) levels and induces N-arachidonyl taurine (NAT) production through interaction with fatty acid binding protein 3 (FABP3), resulting in desensitization of LUAD cells to ferroptosis [164]. In other words, circRNA can interact with microRNA to regulate the corresponding target of ferroptosis and play an anti-ferroptosis role.

Long non-coding RNAs (lncRNAs) are non-coding RNAs with a length of more than 200 nucleotides, which play an important role in many life activities such as regulating energy metabolism, controlling gene expression and protein synthesis [165, 166]. However, more and more studies have identified lncRNAs as key mediators regulating ferroptosis and iron metabolism in lung cancer [167]. LncRNA NEAT1 can target ACSL4 and inhibit the protein expression of ACSL4, thus regulating the sensitivity of lung cancer cells to ferroptosis [96]. Knockdown of lncRNA OGFRP1 can promote lipid peroxidation and iron accumulation in lung cancer, thereby promoting cell ferroptosis. It regulates lung cancer cell proliferation and ferroptosis mainly by inhibiting miR-299-3p and enhancing solute carrier family 38 member 1 (SLC38A1) expression [168]. LncRNA ASMTL-AS1, which is down-regulated in lung cancer cells, can stimulate ferroptosis in lung cancer cells by binding to U2AF2 and stabilizing spermidine/spermine N1-acetyltransferase 1 (SAT1) [169]. LncRNA P53RRA can bind to Ras GTPase activating protein binding protein 1 (G3BP1) to activate the p53 pathway and retain more p53 in the nucleus, thereby exerting a pro-ferroptosis effect [170]. LncRNA C00336 is up-regulated in lung cancer, and it can bind to the RNA-binding protein ELAVL1 to inhibit ferroptosis. Meanwhile, it can also act as an endogenous sponge for microRNA 6852 to regulate the expression of cystathionine β-synthase (CBS), a ferroptosis surrogate marker [171]. While ectopic expression of lncRNA MT1DP can stabilize miR-365a-3p and downregulate Nrf2, sensitizing A549 and H1299 cells to erastin-induced ferroptosis [172]. LncRNA T-UCR Uc.33 is up-regulated in lung adenocarcinoma patients, which can competitively bind to pri-miR-339, inhibit the production of mature miR-339, and further promote the expression of SCL7A11 [173]. In summary, these results suggest that non-coding RNA plays an important regulatory role in ferritin formation in lung cancer. However, the role of non-coding RNA in regulating ferroptosis in lung cancer and its precise mechanism are still much incompletely understood. Therefore, a full understanding of the regulatory process of non-coding RNA in the process of ferroptosis in the occurrence and metastasis of lung cancer, which is of great significance for further elucidating the mechanism of ferroptosis in the process of lung cancer metastasis.

### Other targets

In addition to the regulatory targets described above, there are some ferroptosis targets in lung cancer as follows. N6-methyladenosine (m6A) RNA binding protein YTHDC2 can directly inhibit SLC7A11 on the one hand, and destabilize the mRNA of transcription factor HOXA13 in an m6A dependent manner, thereby inhibiting HOXA13-mediated transcription of SLC3A2, thereby damaging tumor growth. At the same time, it can trigger lipid peroxidation and induce ferroptosis in lung cancer [174]. A20 is a ubiquitin-regulated enzyme, which has considerable application prospect in inflammation and tumors [175]. Small ubiquitin-like modifier (SUMO) specific protease 1(SENP1) can regulate A20 by desumoylation, and then inhibit ferroptosis of lung cancer cells by affecting the interaction between A20 and ACSL4, SLC7A11 [176]. Notch3, the third subtype of the Notch family, can regulate tumor maintenance and affect tumor chemotherapy resistance [177]. Knockdown of Notch3 can increase the ROS level in lung cancer cells, reduce the expression levels of GPX4 and peroxiredoxin 6 (PRDX6), and then induce lipid peroxidation to trigger cell ferroptosis [178]. Ceramide kinase (CERK) inhibits dysregulated voltage-dependent anion-selective channel (VDAC) -mediated mitochondrial function-driven ferroptosis in KRAS mutant lung cancer. Therefore, when CERK is downregulated or inhibited, MMP increases and VDAC modulation occur, sensitizing NSCLC cells with oncogenic KRAS to CDDP [179].

## Drugs targeting ferroptosis in lung cancer

### Chemical drugs

**Fig. 3:**
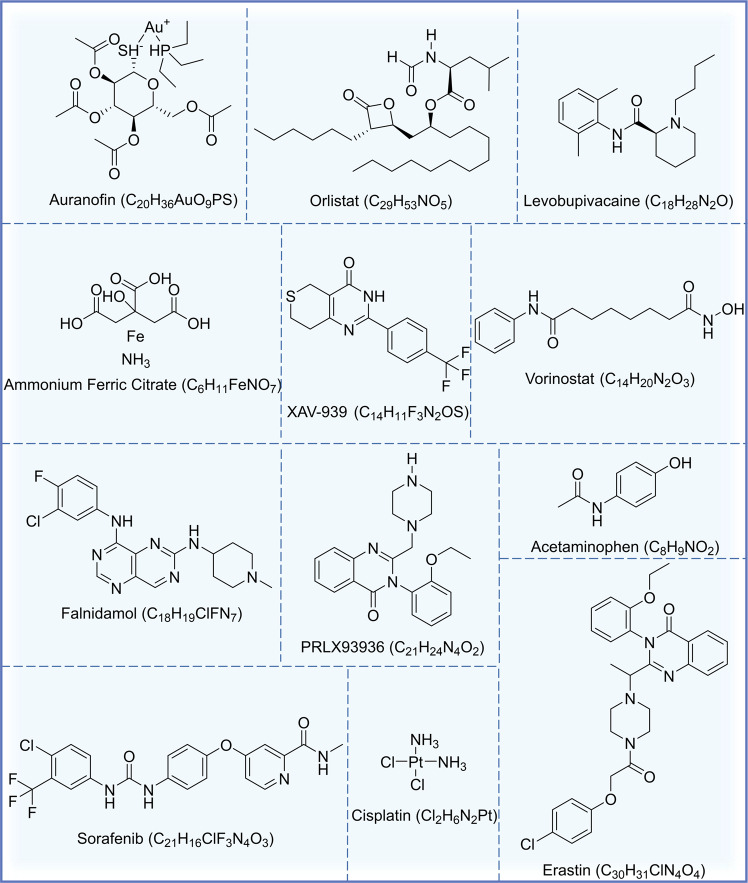
Chemotherapeutic agents acting on ferroptosis in lung cancer. The names, structural formulas, and molecular formulas of these chemical agents that target ferroptosis for lung cancer treatment are shown in the figure.

**Fig. 4:**
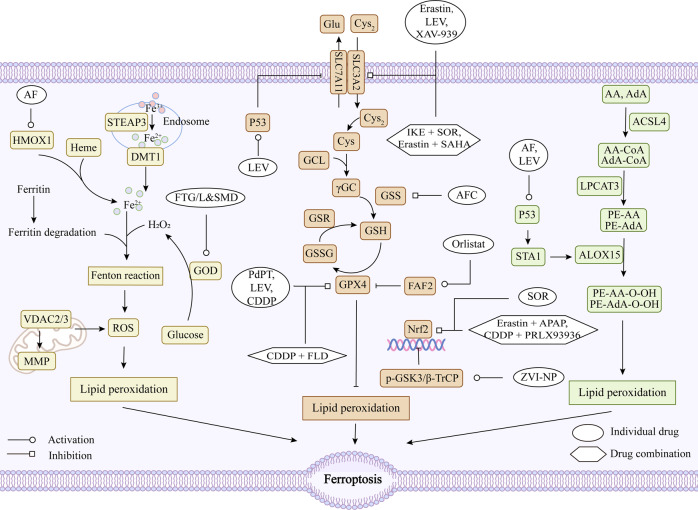
Regulatory mechanisms of chemotherapeutic agents targeting ferroptosis in lung cancer. CDDP Cisplatin, SOR Sorafenib, AF Auranofin, LEV Levobupivacaine, AFC Ammonium Ferric Citrate, PdPT Palladium pyrithione complex, FLD Falnidamol, APAP Acetaminophen, SAHA Vorinostat, IKE Imidazole ketone erastin, ZVI-NP Zero-valent-iron nanoparticle, FTG/L&SMD Multifunctional “ball-rod” Janus nanoparticles, FAF2 fatty acid synthase-associated factor 2, HMOX1 Heme oxygenase 1, GOD glucose oxidase, OH• reactive hydroxyl radical, TrxR thioredoxin reductase 1, DUSP26 Dual-specificity phosphatase 26, GSK3 phospho-glycogen synthase kinase 3, β-TrCP beta-Transducin repeats-containing protein, Nrf2 nuclear factor erythroid 2-related factor 2, p53 tumor suppressor protein, GPX4 glutathione peroxidase 4, System Xc^−^ cystine/glutamate antiporter system, Cys_2_ cystine, Glu glutamate, Cys cysteine, Gly glycine, GCL glutamate-cysteine ligase, SLC3A2 solute carrier family 3, member 2, SLC7A11 solute carrier family 7 (L-type amino acid transporter), member 11, GSS glutathione synthetase, GSH glutathione, GSSG oxidized glutathione, GSR glutathione reductase, ROS reactive oxygen species.

Currently, there are many drugs for tumor ferroptosis, and most of them inhibit system x_c_^−^and GPX4 to promote ferroptosis [53, 54, 180, 181]. The structural formula of the current chemical drugs targeting ferroptosis for lung cancer is shown in Fig. 3. The targets and mechanisms of these chemical drugs regulating ferroptosis for lung cancer are summarized in Fig. 4 and Table 1.

**Table 1:** Chemical drugs targeting ferroptosis in lung cancer.

Drug	Abbreviated name	Test model	Mechanism/Effect	Reference
Cisplatin	CDDP	A549, H358, H460 and Calu-1 cells	Inhibiting the activity of GPX4, promoting GSH depletion and increasing intracellular ROS levels	[192]
Erastin	-	A549, Calu-1, HCC827, H1299 cells, and N5CP cells surgically obtained from patients (N2: Male, 61 years old; N5: male, 58 years old) and nude mouse xenograft models	Upregulating and activating p53, inhibiting Nrf2 and SCL7A11 expression, decreasing cystine uptake, preventing cysteine-dependent GSH synthesis, and inactivating GPX4	[111, 208, 212]
Sorafenib	SOR	A549, H460, H1299 cells and N5CP cells surgically obtained from patients (N2: Male, 61 years old; N5: male, 58 years old) and nude mouse xenograft models	Inhibiting the Nrf2/xCT pathway to regulate glutathione synthesis	[195, 208]
Auranofin	AF	A549, H1975, H2228 and H596 cells	Inhibiting the Trx and GSH systems, leading to a perturbation of redox balance and increasing ROS levels	[196]
Orlistat	-	H1299, A549, LLC cells	Inhibiting the expression of GPX4 and inducing lipid peroxidation	[197]
Levobupivacaine	LEV	A549, A427 cells and and nude mouse xenograft models	Activating p53, inhibiting GPX4 and SLC7A11, and increasing levels of ROS and Fe^2+^	[110]
Ammonium Ferric Citrate	AFC	A549, HCC827, H1299 and H661 cells	Inhibiting GPX4-GSS/GSR-GGT axis activity and increasing Fe^2+^ level and inducing oxidative damage	[198]
Palladium pyrithione complex	PdPT	A549, H1299 cells and nude mouse xenograft models	Promoting GPX4 degradation	[96]
XAV-939	-	H1299 cells	Downregulating SLC7A11	[199]
Zinc	Zn	A549 cells	-	[200]
PRLX93936	-	A549 and H23 cells	Targeting the Nrf2/Keap1 pathway to inhibit GPX4	[203]
Falnidamol	FLD	A549, PC-9 cells and xenograft mouse models	Inhibiting DUSP26, downregulating GPX4, promoting ROS production and mitochondrial dysfunction and inducing lipid peroxidation	[204]
Acetaminophen	APAP	H1299, A549 cells and and nude mouse xenograft models	Regulating Nrf2/HO-1 signaling pathway, reducing glutathione synthesis and increasing lipid peroxide translocation	[206]
Vorinostat	SAHA	HCC827, HCC4006, H1975, H1650, PC9, HCC4011 and H1993 cells	Downregulating SLC7A11	[207]
Imidazole ketone erastin	IKE	Xenograft mouse models and human patient-derived lung adenocarcinoma models	Inhibiting system x_c_^−^ or GPX4	[209]
zero-valent-iron nanoparticle	ZVI-NP	A549, H460, H1299, LLC cells, xenograft mouse models, and spontaneous lung metastasis models	Disrupting the AMPK/mTOR pathway to activate p-GSK3/β-TrCP, degrading Nrf2, and causing mitochondrial dysfunction, intracellular oxidative stress, and lipid peroxidation	[210]
multifunctional “ball-rod” Janus nanoparticles	FTG/L&SMD	-	Combining glucose oxidase (GOD) catalyzes the production of H_2_O_2_ from glucose, generating a highly reactive hydroxyl radical (OH•) via Fenton reaction, leading to lethal peroxide accumulation	[211]

Cisplatin (CDDP) is a traditional platinum-based chemotherapy drug, existing studies have shown that CDDP can induce ferroptosis when the concentration is higher than 5 μg/mL and the action time is longer than 48 h [182]. Sorafenib (SOR) is a novel multi-targeted oral cancer agent that is approved for the treatment of gastrointestinal stromal tumors and metastatic renal cell carcinoma that do not respond to or are not tolerated by standard therapies [183]. But a recent study has shown that SOR also has therapeutic effects on lung cancer and can promote ferroptosis in lung cancer [184, 185]. Olanprofen (AF), an antirheumatic drug, can promote apoptosis by targeting the thioredoxin reductase 1 (TrxR) system, and according to transcriptome analysis, it is also involved in gene regulation of ferroptosis in lung cancer [186]. Orlistat is currently the only OTC weight-loss drug in the world, which can inhibit the progression of lung cancer by targeting FAF2, significantly inhibit GPX4 expression, and induce lipid peroxidation in cells [187]. Levobupivacaine (LEV), a local anesthetic, can inhibit the expression of GPX4 and SLC7A11 by activating p53, thereby exerting a pro-ferroptosis effect and inhibiting the growth of tumors in vivo [104]. Treatment of A549 and HCC827 cells with the food additive ferric ammonium citrate (AFC) for 24 h resulted in a significant decrease in the expression of cell proliferation-related proteins (Ki67, CDK2 and CCND), autophagy-related proteins (ATG3 and LC3A/B) and ferroptosis negative regulators (GPX4 and FTH1), followed by a decrease in intracellular autophagy and induction of cellular ferroptosis [188]. XAV-939, a Tankyrase inhibitor, has been shown to promote ferroptosis in lung cancer cells by inducing the down-regulation of SLC7A11, and inhibiting the progression of lung cancer by down-regulating lncRNA MIR503HG, sponging miR-1273c and regulating SOX4 expression [189].

Compared with the use of a single drug, the existing research trend is to pay more attention to the combination of drugs, on the one hand, to improve the efficacy, on the other hand, to avoid the occurrence of cell resistance. Although cisplatin is a traditional anticancer drug, it is easy to cause cell resistance, which will lead to unsatisfactory anticancer efficacy [190, 191]. PRLX93936, an elastine analogue, can induce lipid peroxidation and ferroptosis in lung cancer cells when combined with CDPP. At the same time, the combination of them can also target Nrf2/Keap1 to regulate the drug sensitivity of lung cancer cells [192]. The combination of faridamol (FLD) and CDPP significantly induced ROS production, free iron accumulation and lipid peroxidation, which greatly triggered ferroptosis [193]. In addition, the combination with ferroptosis inducers is also a current research hotspot. Acetaminophen (APAP) is a traditional antipyretic and analgesic drug [194], which can combine with erastin to promote cell ferroptosis by regulating Nrf2 nuclear translocation and inhibits the growth of lung cancer xenograft tumors [195]. Vorinostat (SAHA), a histone deacetylase inhibitor [196], can inhibit the expression of system x_c_^−^ and significantly enhance the ferroptosis effect of Erastin on EGFR-TKI resistant LUAD cells. Meanwhile, SAHA could block the compensatory upregulation of SCL7A11 induced by Erastin treatment alone and improve the efficacy of the drug [197]. Combination of Erastin or SOR with low-dose CDDP can effectively inhibit the growth of CDDP-resistant NSCLC cells (N5CP) in vivo, and promote ferroptosis by inducing the accumulation of intracellular lipid peroxides [198]. In addition, it has been shown that when the ferroptosis inducer imidazole ketone erastin (IKE), SOR and cytoplasmic proton radiation act synergistically, tumor killing is significantly enhanced in mouse xenograft models and human patient-derived lung adenocarcinoma models [199].

Besides, nanomedicine has also been applied to ferroptosis in lung cancer. A zero-valent-iron nanoparticle (ZVI-NP) can promote ferroptosis in lung cancer cells by enhancing phosphorylation-dependent ubiquitination and Nrf2 degradation, and preferentially accumulate in tumor and lung tissues. At the same time, it can significantly inhibit tumor growth and metastasis, stimulate the in vitro immunity of macrophages and lymphocytes, and weaken the ability of tumor self-renewal [200]. Multifunctional “ball-rod” Janus nanoparticles (FTG/L&SMD) can promote ferroptosis process by initiating the Fenton reaction cycle while effectively inhibiting tumor growth with negligible toxicity in vivo [201].

Therefore, there are still few chemotherapeutic drugs targeting ferroptosis in lung cancer, and most of them are combination drugs. Most of the studies on drugs used alone are drugs originally used for other purposes, and the specific mechanisms of ferroptosis induced by drugs are still less studied. Among the combined drugs, cisplatin and ferroptosis inducer erastin are commonly used, and the synergistic effect with cytoplasmic proton radiation has also been studied. To our surprise, the combination had an excellent anti-drug effect on lung cancer drug resistance. In addition, there are few combined applications with nanotechnology, and its role in promoting ferroptosis of lung cancer is also very impressive.

### Natural medicines

**Fig. 5:**
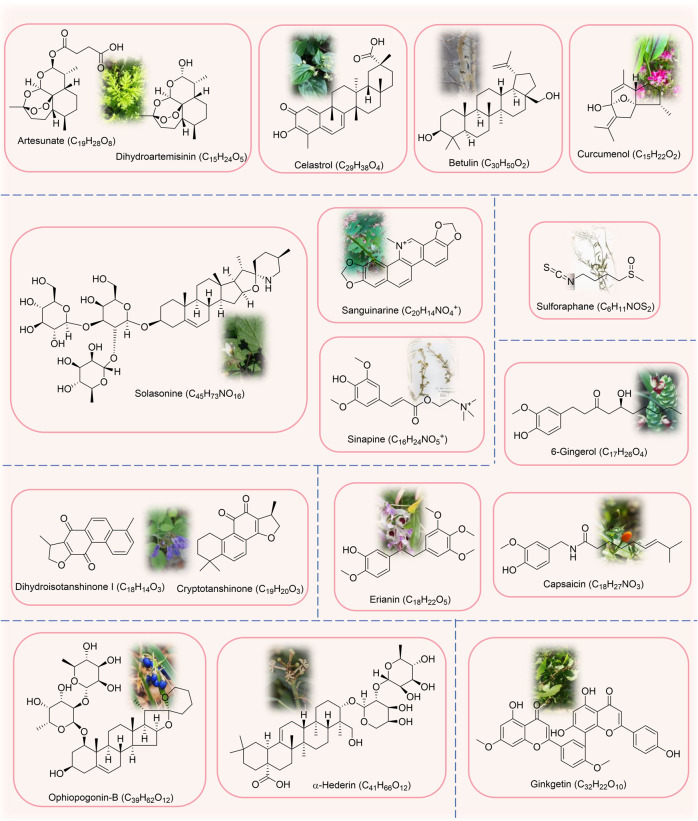
Natural products acting on ferroptosis in lung cancer. Natural products of plant origin that target ferroptosis for lung cancer treatment are shown in the figure and it mainly includes terpenoids, alkaloids, phenols, quinones, saponins, flavonoids, and others.

**Fig. 6:**
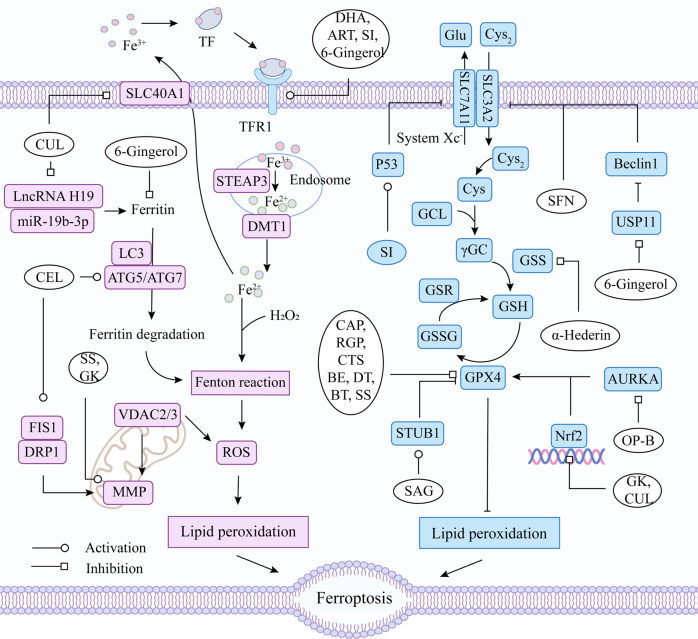
Regulatory mechanisms of natural drugs targeting ferroptosis in lung cancer. ART Artesunate, DHA Dihydroartemisinin, CUL Curcumenol, CEL Celastrol, BE Betulin, SAG Sanguinarine, SI Sinapine, SS Solasonine, CAP Capsaicin, ERI Erianin, DT Dihydroisotanshinone I, CTS Cryptotanshinone, OP-B Ophiopogonin-B, GK Ginkgetin, BT Bufotalin, SFN Sulforaphane, RGP Red ginseng polysaccharide, SLC40A1 solute carrier family 40 member 1, HO-1 Heme oxygenase 1, LC3 microtubule-associated protein 1 light chain, ATG5 autophagy-related protein 5, ATG7 autophagy-related protein 7, PRIM2 DNA primase polypeptide 2, FIS1 mitochondrial adaptor fission 1, DRP1 dynamin related protein 1, FTH1 ferritin heavy chain 1, AURKA Aurora kinase A, USP11 ubiquitin-specific processing protease 11, MMP mitochondrial membrane potential, miRNA MicroRNA, lncRNA Long non-coding RNA, DMT1 solute carrier family 11 member 2, Nrf2 nuclear factor erythroid 2-related factor 2, p53 tumor suppressor protein, GPX4 glutathione peroxidase 4, System Xc^−^ cystine/glutamate antiporter system, Cys_2_ cystine, Glu glutamate, Cys cysteine, Gly glycine, GCL glutamate-cysteine ligase, SLC3A2 solute carrier family 3, member 2, SLC7A11 solute carrier family 7 (L-type amino acid transporter), member 11, GSS glutathione synthetase, GSH glutathione, GSSG oxidized glutathione, GSR glutathione reductase, ROS reactive oxygen species.

Natural compounds have also been shown to be useful in the treatment of cancer ferroptosis due to their rich biological activities [202]. Currently, the natural compounds for ferroptosis in lung cancer mainly include terpenoids, alkaloids, phenols, quinones and saponins, and also contain few steroids and flavonoids. Figure 5 lists the sources and chemical formulas of natural products currently acting on ferroptosis for lung cancer treatment, and their regulatory targets and mechanisms are shown in Fig. 6 and Table 2.

**Table 2:** Natural medicines targeting ferroptosis in lung cancer.

Classification	Compound	Abbreviated name	Origin	Test models	Mechanism/Effect	Reference
Terpenoids	Artesunate	ART	*Artemisia annua* L.	H1299, A549, LTEP-a-2, H23 and H358 cells	Inhibiting system x_c_^−^, up-regulating transferrin receptor (TFR) levels, and inductng ROS production	[219]
Terpenoids	Dihydroartemisinin	DHA	*Artemisia annua* L.	H1299, A549, LTEP-a-2, H23, H358, XWLC-05, LLC cells and nude mouse xenograft models	Inhibitioing PRIM2/SLC7A11 axis, GPX4, up-regulating the level of transferrin receptor (TFR) and decreasing the level of GSH	[219–221]
Terpenoids	Curcumenol	CUL	*Curcuma zedoaria* (Christm.) Roscoe	H1299, H460 cells and xenograft mouse models	Down-regulating lncRNA H19 targeted miR-19b-3p to regulate the level of FTH1, increasing the expression levels of heme oxygenase-1 (HO-1) and transferrin (TF), and reducing the levels of GPX4, SLC40A1, SLC7A11, FTH1 and Nrf2	[223]
Terpenoids	Celastrol	CEL	*Tripterygium wilfordii* Hook.fil.	HCC827, A549, H1299 cells and nude mouse xenograft models	Promoting ROS production, disrupting mitochondrial membrane potential, enhancing the interaction between dynamin-related protein 1(DRP1) and mitochondrial fission 1 protein (FIS1), and promoting mitochondrial fission	[212]
Terpenoids	Betulin	BE	*Betula platyphylla* Sukaczev	A549, H460 cells and xenograft mouse models	Inducing ROS accumulation, lipid peroxidation GSH depletion, and inducing the ferroptosis-related gene expression	[226]
Alkaloids	Sanguinarine	SAG	*Macleaya cordata* (Willd.) R.Br.	A549, H3122 cells and xenograft mouse models	Reducing the protein stability of GPX4 by ubiquitination and endogenous GPX4 degradation mediated by the E3 ligase STUB1	[228]
Alkaloids	Sinapine	SI	Cruciferae	H460, H661, A549 cells and xenograft mouse models	Upregulating TF and TFR, downregulating SLC7A11 in a p53-dependent manner and increasing intracellular Fe^2+^, lipid peroxidation, and ROS levels	[230]
Alkaloids	Solasonine	SS	*Solanum nigrum* L.	Calu-1 and A549 cells	Inhibiting the expression of SLC711 and GPX4, depleting GSH and Cys, promoting the hyperpolarization of mitochondrial membrane potential (MMP) and causing redox imbalance and mitochondrial dysfunction	[232]
Phenols	6-Gingerol	-	*Zingiber officinale* Roscoe	A549 cells and nude mouse xenograft models	Inhibiting USP14-mediated deubiquitination of Beclin1 at K6, up-regulating the expression of Beclin-1, LC3I, LC3II, NCOA4 and TFR and inhibiting the expression of FTH1	[234]
Phenols	Capsaicin	CAP	*Capsicum annuum* L.	A549 and H23 cells	Inhibiting SLC7A11/GPX4 signaling	[237]
Phenols	Erianin	ERI	*Dendrobium nobile* Lindl.	H1299, H460 cells and nude mouse xenograft models	Activating CAM and regulating L-type voltage-dependent Ca^2+^ channels (LVDCC), leading to increased Ca^2+^ uptake, ROS production and Fe^2+^ levels	[238]
Quinones	Dihydroisotanshinone I	DT	*Salvia miltiorrhiza* Bunge	A549, H460 cells and nude mouse xenograft models	Inhibiting GPX4	[241]
Quinones	Cryptotanshinone	CTS	*Salvia miltiorrhiza* Bunge	A549 and H520 cells	Inhibiting GPX4, inducing ROS production and iron-dependent lipid peroxidation	[243]
Saponins	Ophiopogonin-B	OP-B	*Ophiopogon japonicus* (Thunb.) Ker Gawl.	A549 cells and nude mouse xenograft models	Inhibiting AURKA, down-regulating the protein expressions of GPX4, xCT, FTL and FTH1, and up-regulating the protein expressions of PTGS2 and ACSL4	[246]
Saponins	α-Hederin	-	*Hedera nepalensis* K.Koch	A549, PC9 cells, nude mouse xenograft models and lung metastasis models	Interacting with GSS and GPX2 through hydrogen bonding, down-regulating GPX2 and GSS expression, inhibiting GSH synthesis, and disrupting the GSH redox system	[248]
Flavonoids	Ginkgetin	GK	*Ginkgo biloba* L.	A549, H460, SPC-A-1 cells and nude mouse xenograft models	Inhibiting Nrf2/HO-1, down-regulating the expression of SLC7A11 and GPX4, increasing ROS levels, and promoting MMP loss	[250]
Steroids	Bufotalin	BT	Venenum Bufonis	A549 cells and nude mouse xenograft models	Inhibiting GPX4, promoting ubiquitination and degradation of GPX4, promoting the production of lipid ROS, and increasing intracellular Fe^2+^	[252]
Others	Sulforaphane	SFN	Cruciferae	H69, H69AR and H82 cells	Inhibiting SLC7A11, decreasing GSH levels and increasing ROS levels	[255]
Others	Red ginseng polysaccharide	RGP	*Panax ginseng* C.A.Mey.	A549 cells	Downregulating GPX4, promoting the release of LDH and accumulation of lipid ROS	[257]

#### Terpenoids

Artemisinin and its derivatives are well known for their ability to treat malaria [203], but more and more studies have shown that artemisinin is also effective in promoting tumor ferroptosis [204, 205]. Artesunate (ART) and dihydroartemisinin (DHA), derivatives of artemisinin [204, 206], can induce ROS-dependent ferroptosis, down-regulate the protein level of voltage-dependent anion channel (VDAC), and promote cell apoptosis [207]. DHA can down-regulate PRIM2 and regulate the expression of SLC7A11 and β-catenin, thereby inhibiting the proliferation and cloning ability of lung cancer cells and promoting ferroptosis [208]. In addition, DHA can also combat the resistance of lung cancer cells to chlorinated protein e6 (Ce6) -mediated photodynamic therapy (PDT) by promoting ferroptosis [209]. Curcuma (CUL) is a terpenoid compound derived from *Curcuma zedoaria* (Christm.) Roscoe [210], which can promote the ferroptosis process of lung cancer in vitro and in vivo, and has good anticancer effect. lncRNA H19 can act as a competitive endogenous RNA to bind to miR-19b-3p, thereby enhancing the transcriptional activity of its endogenous target ferritin heavy chain 1(FTH1), and CUL plays a role in promoting ferroptosis by down-regulating lncRNA H19 in lung cancer cells [211]. Celastrol (Cel), a natural triterpenoid compound [212], can increase the production of reactive oxygen species (ROS), disrupt mitochondrial membrane potential, promote mitochondrial fission, and further activate ATG5/ ATG7-dependent autophagy, and inhibit tumor growth in vivo when combined with erastin [213]. Betulin (BE) is a triterpenoid compound in the bark of *Betula platyphylla* Sukaczev [214], which can overcome gefitinib resistance in EGFR wild-type /KRAS mutant NSCLC cells by inducing ferrodeath when used in combination with gefitinib [215].

#### Alkaloids

Sanguinarine (SAG), an isoquinoline alkaloid, can promote the production of reactive oxygen species and inhibit the JAK/STAT pathway to promote apoptosis of lung cancer cells [216]. In addition to this, treatment of A549 and H3122 cells with SAG caused an increase in intracellular Fe^2+^ concentration, ROS levels and MDA content as well as a decrease in GSH content, which was thus shown to also trigger ferroptosis in lung cancer [217]. Sinapine (SI), an alkaloid isolated from rapeseed and cruciferous plants, targets mitochondria to regulate mitochondrial oxidative stress and induces ferroptosis in lung cancer cells [218, 219]. Solanine (SS), a compound derived from *Solanum nigrum* L. Steroid glycoside alkaloids [220], which can cause iron overload and redox imbalance in Calu-1 and A549 cells, and then trigger ferroptosis [221].

#### Phenols

6-gingerol is a monophenolic compound derived from rhizome of *Zingiber officinale* Roscoe [222], which can promote ferroptosis of lung cancer in vitro and in vivo by inhibiting the expression of autophagy-related protein ubiquitin-specific peptidase 14 (USP14) and regulating the downstream of autophagy-dependent ferroptosis [223]. Capsaicin (CAP), a natural active ingredient in *Capsicum annuum* L. and the source of their pungent taste, has anticancer activity [224, 225]. In the ferroptosis related studies of lung cancer, CAP can inhibit the proliferation of A549 and NCI-H23 cells and induce ferroptosis by inactivating SLC7A11/GPX4 signaling pathway [226]. Erianin, an extract from *Dendrobium nobile* Lindl. can promote G2/M cell cycle arrest, inhibit cell migration and induce ferroptosis in lung cancer cells, while inhibiting tumor growth in vivo [227, 228].

#### Quinones

Dihydroisotanshinone I (DT) is a bioactive compound derived from the rhizome of *Salvia miltiorrhiza* Bunge [229], which can inhibit the growth of lung cancer cells, promote the apoptosis and ferroptosis of lung cancer cells, and inhibit the metastasis of A549 cells in vivo [230]. Meanwhile, another kind of cryptotanshinone (CTS) extracted from the rhizome of *Salvia miltiorrhiza* Bunge can not only prevent pulmonary fibrosis [231], but also promote ferroptosis of lung cancer by inhibiting GPX4 activity, and activate caspase-3 to promote apoptosis of lung cancer [232].

#### Saponins

Ophiopogon B (OP-B) is a saponin compound derived from the root of *Ophiopogon japonicus* (Thunb.) Ker, and its inhibitory effect on lung cancer has been studied to some extent [233, 234]. According to proteomic sequencing analysis, AURKA, a classical cell cycle regulatory protein kinase, is highly expressed in NSCLC, and OP-B promotes ferroptosis in lung cancer in vitro and vivo by targeting AURKA [235]. α-heguelin is a monodesmotic triterpenoid saponin isolated from *Hedera nepalensis* K.Koch [236], which induces apoptosis at high doses, disrupts the redox system by significantly affecting glutathione metabolism at safe and low toxic doses to induce ferroptosis in NSCLC, and also increases the sensitivity of NSCLC cells to cisplatin [237].

#### Others

Ginkgetin (GK), a diflavone isolated from *Ginkgo biloba* L. [238], when combined with cisplatin, can induce Nrf2/HO-1 inactivation, increase unstable iron pool and lipid peroxidation, on the one hand, promote ferroptosis in lung cancer, on the other hand, significantly enhance the therapeutic effect [239]. Bufotalin (BT) is a steroid lactone derived from bufalin, which has anti-tumor activity [240]. It can not only promote ferroptosis of lung cancer cells in vitro, but also inhibit the growth process of xenograft tumors in vivo [241]. Sulforaphane (SFN) is a natural isothiocyanate present in a variety of vegetables [242], which can target Nrf2 and has potential anti-cancer activity [243]. At the same time, SFN can also inhibit the expression of SLC7A11, leading to the decrease of GSH and the increase of ROS level in lung cancer cells [244]. Red ginseng polysaccharide (RGP) is the active ingredient in *Panax ginseng* C.A.Mey. [245], which can induce the release of lactate dehydrogenase (LDH) in A549 cells, inhibit the expression of GPX4, and play a role in promoting ferroptosis [246].

### Chinese medicine preparations

In addition, some studies have shown that some Chinese medicine preparations can also have potential therapeutic effects on lung cancer by promoting ferroptosis. For example, hedyotis diffusa injection can induce ferroptosis in lung cancer by inhibiting Bcl2, promoting Bax and activating VDAC2/3 channel, thereby increasing the release of ROS [247]. Fuzheng Kangai Decoction (FZKA) can induce ferroptosis of lung cancer cells by inhibiting GPX4, increasing lipid peroxidation and cellular Fe^2+^ level, and inhibit tumor growth in vivo [248]. Furthermore, there are no more studies on the ferroptosis of lung cancer about Chinese medicine preparations, which is also a research blank at present. How to combine traditional Chinese medicine compound with new programmed death pathway to overcome the shortcomings of lung cancer treatment is a problem that many researchers of Chinese medicine preparations need to think about.

## Conclusions and perspectives

Targeting the cell death process is a common approach in cancer therapy, and ferroptosis, a new form of programmed cell death, is also thought to play an important role in anti-cancer treatment. Ferroptosis, a hot topic of research in recent years, has been gradually enriched by its corresponding regulatory mechanisms. Nevertheless, there are still many research gaps regarding the regulatory mechanism of ferroptosis, which needs to be further explored and solved in future ferroptosis-related studies. Also, the increased reactive oxygen species-induced lipid peroxidation response exhibited as a result of the regulation of ferroptosis is present in other cell death pathways [249], How to clarify that the cells undergo ferroptosis and find more exclusive physiological and biochemical indicators of ferroptosis is the focus in current research.

In contrast, in the existing lung cancer studies, the targets related to ferroptosis are more often targeted to the system x_c_^−^ or GPX4 system, and there is a lack of studies on non-classical GPX4 regulatory pathways, with only few studies on FSP1. There exist some studies on Nrf2, a key factor of oxidative stress, but on the one hand, Nrf2 has preventive oxidative and anti-inflammatory effects on normal cells, on the other hand, it can promote tumor metastasis and induce drug resistance [250, 251]. In addition, the tumor suppressor P53 also plays a regulatory role in ferroptosis of lung cancer by inhibiting the expression of SLC7A11, the key component of system x_c_^−^ [101]. However, it has also been shown that P53 inhibits erastin-induced ferroptosis by blocking dipeptidyl peptidase-4 (DPP4) activity in a transcriptionally non-dependent manner [252]. How to correctly view the role of Nrf2 and P53 in ferroptosis of lung cancer is to be investigated. What’s more, there are a few studies related to NFS1, STYK1, LSH and other regulatory targets, but the relevant research content is not in-depth. It is important to mention that studies promoting ferroptosis by targeting iron metabolism and lipid peroxide formation are clearly lacking. Meanwhile, non-coding RNAs affect the expression level of target genes through the competitive endogenous RNA regulation mechanism, and also have an appreciable regulatory role in lung cancer ferroptosis [253]. Furthermore, it is worth noting that although ferroptosis is a new programmed cell death pathway, many studies define ferroptosis as an autophagy-dependent death pathway, which has some potential connection with autophagy [133, 135, 213, 254, 255], and there is also a good research prospect to explore the regulatory relationship between ferroptosis and autophagy.

In the research of drugs that promote tumor ferroptosis, most of them also target system x_c_^−^ or GPX4 drugs [256]. However, the existing researches on ferroptosis in lung cancer mainly focus on chemical drugs, natural drugs and the combination of drugs and drugs, and there is also a small amount of synergistic effect of drugs with certain therapies. Fortunately, some drugs can not only promote ferroptosis, apoptosis, induce cell cycle arrest or inhibit cell growth and migration in vitro, but also inhibit tumor progression in vivo. At the same time, when the two drugs are combined or a drug combined with a certain therapy, it can also play a better therapeutic effect and resist lung cancer drug resistanc. Compared with chemical drugs, there are more studies on natural drugs in lung cancer ferroptosis, and the regulatory pathways involved are broader, which also indicates that natural compounds have considerable research value in lung cancer ferroptosis. However, it cannot be ignored that although ferroptosis has a positive regulatory effect on tumors, it is extremely unfavorable for normal cells. In the cardiovascular system and nervous system, inhibition of ferroptosis is required to play a protective role [257, 258]. How to selectively apply ferroptosis to the treatment of lung cancer, combat the drug resistance and radiation resistance of lung cancer, inhibit the development and metastasis of lung cancer, and protect normal cells from ferroptosis is a difficulty in the future research of ferroptosis in tumors.
